# Virtual sarcoma disease multidisciplinary team: a successful experience in the era of telemedicine and COVID-19 in Italy

**DOI:** 10.3389/fpubh.2025.1636095

**Published:** 2025-11-06

**Authors:** Francesca Salvatori, Barbara Rossi, Concetta Elisa Onesti, Sabrina Vari, Serena Ceddia, Elisa Checcucci, Antonella Cosimati, Federica Riva, Davide Renna, Roberto Biagini, Virginia Ferraresi

**Affiliations:** 1 Medical Oncology, University of Rome “La Sapienza”, Rome, Italy; 2 Oncological Orthopaedics Unit, IRCCS Regina Elena National Cancer Institute (IRE), Rome, Italy; 3 UOSD Sarcomas and Rare Tumours, IRCCS Regina Elena National Cancer Institute (IRE), Rome, Italy; 4 Oncology and Oncohematology Unit, Regional General Hospital “F. Miulli”, Bari, Italy

**Keywords:** COVID-19, sarcoma, telemedicine, disease multidisciplinary team, virtual disease multidisciplinary team

## Abstract

**Background:**

Due to their rarity and complexity sarcomas require specialized multidisciplinary team management. COVID-19 pandemic brought to a rapid implementation of telemedicine and activation of digital tools. This study evaluates the perception of virtual disease multidisciplinary team among healthcare professionals of an European rare cancer referral center.

**Methods:**

An online survey was administered to the participants of Regina Elena National Cancer Institute’s Sarcoma disease multidisciplinary team meetings held between 2020 and 2022. It was composed of 40 questions comparing face-to-face and virtual meetings. Data from the Institutional disease multidisciplinary team from 2019 to 2022 were also analyzed retrospectively to compare the pre-covid, covid, and post-covid phases.

**Results:**

Twenty-two healthcare professionals answered the survey. In their opinion, decision-making process was not affected by virtual modality (86.0%). Regarding virtual meetings 90.0% were highly/moderately satisfied with depth of discussion, 95.0%–100% were able to interact adequately and access all relevant data. The most important improvements of virtual disease multidisciplinary team were better quality of clinical approach/research (22.7%–31.8%), technological innovations (50.0%), and logistical setting (95.5%). 90.0% to 100% thought that virtual disease multidisciplinary team could be approved thereafter. We observed an increase in participation rate from 58.0, to 62.0%, to 64.0% (*p* = 0.0159) and a rise in the new cases discussed at meetings compared to the re-discussed ones from 30.1% to 37.9% to 42.3% (*p* < 0.0001) in the pre-covid, covid, and post-covid phases, respectively.

**Conclusion:**

Virtual disease multidisciplinary team enhances participation and discussion quality without compromising patient care.

## 1 Introduction

Sarcomas are extremely rare malignant tumors and represent less than 1% of all new malignancies’ diagnosis (1). The 5th edition of World Health Organization (WHO) classification recognizes more than 80 different histological types of sarcomas categorized as soft tissue (STS) or bone sarcomas (BS) (2).

STS incidence increases with age, with a peak between 80 and 89 years and a mean age of diagnosis around 60 years. BS have a bimodal age distribution, with peaks between ages 10 and 30 and 60 and 90 (1).

In Europe, the incidence of STS is 4–5 new cases/100,000 people, which is six times more common than BS, whose incidence is around 0.8 new cases/100,000 people (3). In Italy, in 2021 about 2,300 new cases were detected (1,400 among men and over 900 in women), with a prevalence of about 29,000 people living with a diagnosis of sarcoma (4).

Besides being rare, they are also sneaky tumors as they are ubiquitous with often non-specific symptoms and consequently a diagnosis more complex and challenging, often leading to a delay (5–7). Prognosis varies greatly and European countries follow different indications on trying to establish the best-possible care. Many countries have their own sarcoma networks and collaborate on several European-funded research projects. In Italy, cancer treatment is performed in various general hospitals or cancer centres throughout the country, but most of these are not highly specialised in the treatment of sarcomas, which are therefore referred to centres recognised by European Reference Network on Rare Adult Cancers (EURACAN) (8–10).

Disease multidisciplinary team (DMT) meetings represent a crucial step in decision-making process, with the aim of defining an optimal therapeutic strategy in relation to shared guidelines (11). Nowadays it constitutes the standard of care as it optimizes the coordination between all the necessary professionals involved in patients’ care and several national guidelines (12). DMT should be composed by a core of experts including all the necessary key professionals: surgeon with experience in cancer treatment, medical oncologist, radiologist, pathologist, radiotherapist, case manager, and data manager. Other professionals both internal to the institute and external consultants can be involved, according to the needs and the complexity of the individual case discussion (13). Multidisciplinary meetings make the clinician feel more confident with the therapeutic decision, significantly shortens the interval from diagnosis to treatment, leads to a better patient experience and an increase in the quality of care for patients (14). Some evidences suggest that implementing DMT discussions improves patients’ outcomes and reduced cancer-related mortality, although it remains difficult to evaluate the real effect on survival (15–17). All studies concluded that a multidisciplinary setting resulted in improved patient outcomes in terms of diagnosis and/or treatment planning, improvements in survival, patient satisfaction, and clinician satisfaction as a consequence of teamwork communication and cooperation (18).

The COVID-19 pandemic due to SARS-CoV-2 infection has imposed a sudden and rapid reorganization in care management and affected the professional life of the entire healthcare sector, including the Centres dedicated to the treatment of oncological diseases (19–23). This resulted in the adherence of all health professionals to the preventive measures recommended by WHO and government measures, including social distancing, logistical limitations, and restrictions on interpersonal exchanges (24). It also gave the opportunity to activate digital health tools such as the transition from face-to-face (FTF) DMT meetings to virtual ones (vDMT).

The aim of this study is to examine and describe the experience of the Institutional Sarcoma DMT in carrying out virtual meetings, comparing this new modality with the previous FTF one, and to investigate how DMT participants perceive the introduction of the virtual modality by administering a survey to them. Some aspects in terms of advantages, disadvantages and effectiveness of virtual modality are discussed in order to see if the adoption of this procedure is valuable in the decision-making process and in amelioration of clinical practice.

## 2 Materials and methods

We administered an online survey (Table 1) to the participants of the biweekly Sarcoma DMT meetings held at Regina Elena National Cancer Institute of Rome, an EURACAN referral centre.

**Table 1:** Online survey.

Experience of individual professional in MDT
1. Name:
2. Professional role:
3. If you are Medical Doctor or Resident, what is your primary specialty?
4. If you answer other specialty at question 3, please specify:
5. How many years have you been in this specialty?
6. Are you part of the Healthcare Staff of IRCCS Regina Elena National Cancer Institute?
7. How many years have you been involved in Oncology?
8. From which year have you been participating in Sarcomas DMT meetings at IRCCS Regina Elena National Cancer Institute?
9. Do you join any other DMT in your/our Institute besides Sarcomas DMT?
10. If yes question 9, which are they (specify specialty of DMT)?
11. Besides Sarcomas DMT at our Institute, do you join any other Sarcomas/other specialty DMT in other Cancer Centres?
12. If Yes question 11, specify specialty of DMT and other Cancer Centres
13. Have you participated in any virtual DMT meeting before this experience?
14. If Yes question 13, how many?
About FTF DMT
15. What was your opinion regarding the conventional face-to-face DMT meeting?
16. What are the most important advantages of a face-to-face DMT meeting? (one or more options accepted)
17. Other comments on question 16:
18. What are the disadvantages of a face-to-face DMT meeting? (one or more options accepted)
19. Other comments on question 18:
20. If you have attended a face-to-face multidisciplinary meeting in other Institutes (questions 11–12), could you notice any differences in terms of organization and/or development, compared to DMT meetings in our Institute?
21. If yes question 20, please specify advantages and/or disadvantages:
22. Did you have to join face-to-face DMT meetings at the early onset of COVID-19 pandemic?
23. If yes question 22, which preventive measures were encouraged among them? (one or more options accepted)
24. If no question 22, did you intentionally avoid it or did it not happen to you?
About virtual MDT
25. What was your opinion initially, when the Institute’s Scientific Direction Board implemented virtual DMT meetings because of the COVID-19 pandemic?
26. Do you think the decision-making process to achieve a diagnosis has been affected due to the switch to virtual meeting modality?
27. Do you think there has been an increase in “change in treatment plan” in virtual DMT meetings compared to conventional ones?
28. Were you able to interact adequately with other specialists and members in the virtual meeting?
29. Were you able to access all relevant patient data (images, clinical details, histology, etc.) in the virtual meeting?
30. Do you think adequate expertise of specialists was available in the health-care decision making process?
31. Do you think you have adequate time for discussion of cases in virtual modality?
32. Are you satisfied with technical support during virtual meetings (availability of microphone, head/earphones, signal speed, intranet Wi-Fi, virtual platform, file sharing, etc.)?
33. Are you satisfied overall with the depth of discussion during virtual meetings, compared with conventional face-to-face ones?
34. Has your DMT meetings attendance frequency changed in virtual setting compared to face-to-face?
35. In relation to multidisciplinary approach, do you think the COVID-19 pandemic has affected cancer patients’ care?
Future perspectives
36. According to your current experience, do you think virtual DMT meetings could be approved hereafter?
37. Do you think virtual DMT meetings will be the future in treatment of Cancer care?
38. Do you think virtual modality will facilitate to expand DMT meetings globally (multicentric committee meetings, complex cases sharing among Specialists from other centers worldwide)?
39. In your opinion, what kind of changes has virtual mode brought? (One or more options accepted)
40. Please, add any other comment in relation to question 39 and future perspective on the topic.

DMT, disease multidisciplinary team; FTF, face-to-face.

We formulated 40 questions, divided in four sections: the first section (14 questions) was about the experience of individual professional in DMT; the second section (10 questions) asked opinions about FTF meetings; the third (11 questions) about the virtual one; the last section (5 questions) inquires about the future perspectives of the online modality. Questions contemplated 12 yes/no answers, 15 multiple choice answers with one or more items to be selected, two 5-points Likert scale, and 11 open answers. The survey was conceived by one medical doctor participating in DMT meetings and independently reviewed by three other medical doctors participants in sarcoma DMT meetings, before starting the study.

We also analysed in a retrospective way the registries of all the DMT meetings held from 9 March 2019 to 8 March 2022 including data about numbers of participants, numbers of external participants, and numbers of discussed cases (new cases vs. known cases discussed several times).

We performed a descriptive statistic for the survey responses. Subsequently, we analysed data from the institutional DMT. We defined three different time periods by using the date of National lockdown beginning in Italy (9 March 2020) as cutoff: a pre-covid phase extending from 9 March 2019 to 8 March 2020; a covid phase extending from 9 March 2020 to 8 March 2021, and post-covid phase extending from 9 March 2021 to 8 March 2022. We analysed all the data from the institutional DMT focusing on time periods, without calculating a sample size.

We checked normality by Kolmogorov–Smirnov test for all the continuous variables. The study groups were compared using Kruskal–Wallis test, followed by Dunn’s multiple comparison test, or Mann–Whitney U test as appropriate. The number of new cases and re-discussed cases per time-period were analysed by Chi Square test. Statistical analysis was performed by means of Prism GraphPad v.9 software, Insightful Science.

## 3 Results

### 3.1 Survey results

A total of 22 out of 25 healthcare professionals participating to the DMT, corresponding to a response rate of 88.0%, have answered to the survey: seven orthopaedics, four oncologists, one radiotherapist, two pathologists, one radiologist, one nuclear medicine physician, one psychologist, two residents in training, two data managers, and one case manager. Thirteen of the participants have been working in their specialty for more than 10 years, seven between 5 and 10 years, and two for less than 5 years. Thirty-six percent of them usually join other DMT meetings (otorhinolaryngology, melanoma, thyroid, breast, hematologic, gastrointestinal, head & neck, and gynecologic DMT); 18.0% had already participated in a virtual meeting before this experience.

**Figure 1:**
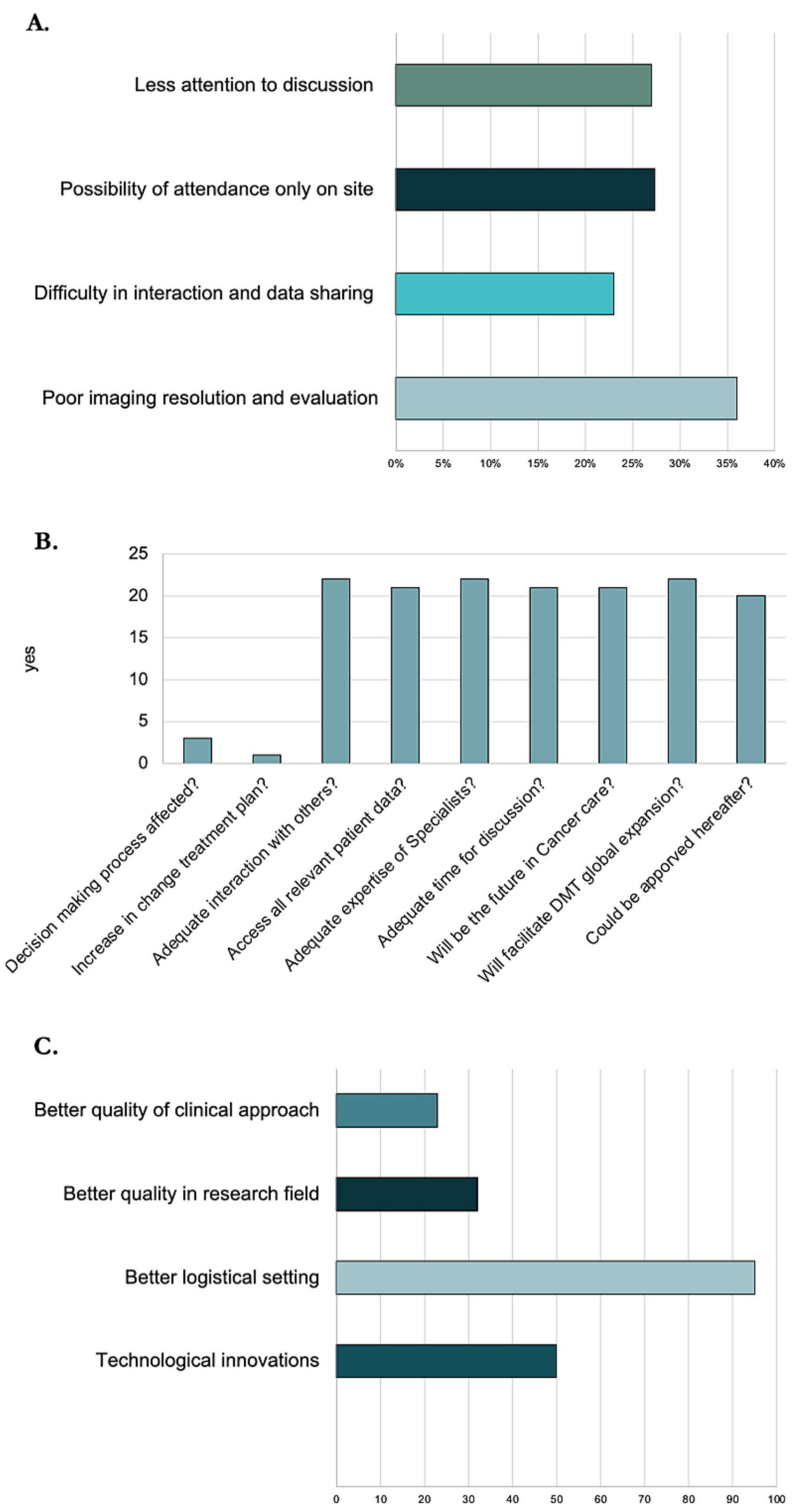
Survey data about opinion on virtual and face-to-face DMT. **(A)** Bar chart representing disadvantages of face-to-face DMT. **(B)** Bar chart representing opinions about virtual DMT. **(C)** Bar chart representing changes that virtual DMT has brought.

Overall, 91.0% answered that FTF modality was good or very good and that the most important advantages were the facilitation in interaction with other participants (68.0%) and greater attention to discussion because of the predefined duration (50.0%). However, the disadvantages observed by the participants were various: less attention to discussion for 27.3% of the participants; the possibility of attendance only on site for 27.3%; the difficulty in interaction and data sharing for 22.7%; and the poor imaging resolution and evaluation for 36.4% (Figure 1A).

About the vDMT (Figure 1B): 86.0% thought that the decision-making process had not been affected and 95.0% stated that there was not a change in the treatment plans; 100% were still able to interact adequately with other specialists and members and to access all relevant patient data (images, clinical details, histology, etc.) and affirmed that an adequate expertise of specialists was available in the decision-making process. For the majority (95.0%) the time available for the discussion of cases was adequate.

In general, 90.0% were highly or moderately satisfied overall with the depth of discussion and above all with the technical support (availability of microphone, head/earphones, signal speed, intranet Wi-Fi, virtual platform, file sharing, etc.). Participants declared that the attendance at the meetings with the new modality was similar (68.0%) or increased (32.0%).

In the opinion of the participants, the most important change that the virtual mode brought was better quality of clinical approach (22.7%) and of the research field (31.8%). Moreover, there has been a notable important technological innovation (50.0%), and a better logistical setting, like the possibility to connect outside the hospital (95.5%) (Figure 1C).

Finally, 91.0% thought that vDMT could be approved hereafter and 100% stated that it would facilitate the global expansion of DMT meetings (multicentre committee meetings, complex case sharing among specialists from other centres worldwide).

### 3.2 Institutional DMT data

**Figure 2:**
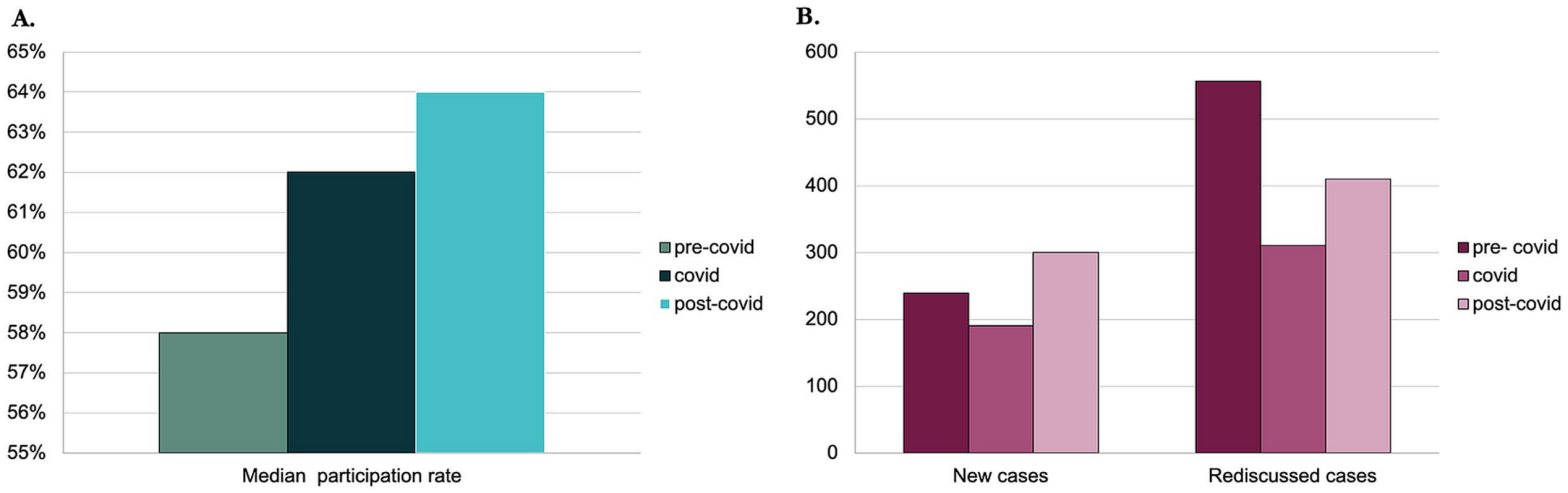
Data from Institutional DMT. **(A)** Bar chart representing the rise of participation rate between the pre-covid, covid, and post-covid periods. **(B)** Bar chart representing the number of new cases and re-discussed ones between pre-covid, covid, and post-covid periods.

Analysing data from the Institutional DMT (Table 2), in the 12-months pre-covid period we held 84 meetings, and the median number of participants was 11 (range: 6–20) over 20 members and we had only six external guests in a few meetings. During the 12-months covid period we held 51 meetings with a median number of participants of 14 (range: 4–23) over 22 members with no external guests. In the 12-months post-covid period we held 93 meetings, and the median number of participants was 16 (range: 7–26) over 25 members and we had 11 external guests in many meetings. The median proportion of participation (Figure 2A) in the three different periods was, respectively, in the pre-covid, covid, and post-covid periods of 58.0% (95% CI 55.0%–60.0%), 62.0% (95% CI 48.0%–71.0%), and 64.0% (95% CI 61.0%–68.0%), with an increase in participation rate statistically significant from the pre-covid to the post-covid period (*p* = 0.0159). The multiple comparison analysis showed that only the difference between the median participation rate during pre- and post-covid period was statistically different (*p* = 0.0127).

**Table 2:** Institutional DMT data in pre-covid, covid, and post-covid period.

	Pre-covid period	Covid period	Post-covid period
Total number of meetings	84	51	93
Median number of participants per meeting	11 (6–20)	14 (4–23)	16 (7–26)
Number of members	20	22	25
Total number of external guests	6	0	11
Proportion of participation	58% (95% CI 55–60%)	62% (95% CI 48–71%)	64% (95% CI 61–68%)
Total number of discussed cases	795	501	710
Median number of cases per meeting	9 (2–20)	10 (1–17)	6 (1–22)
Total number of NC	239 (30.06%)	190 (37.92%)	300 (42.25%)
Total number of RD	556 (69.94%)	311 (62.08%)	410 (57.75%)
Median number of NC per DMT	2 (0–10)	3 (0–12)	2 (0–12)
Median number of RD per DMT	6 (0–15)	5 (0–12)	4 (0–14)

Data from the Institutional DMT in the 12-months pre-covid, covid, and post-covid period. DMT, disease multidisciplinary team; NC, new discussed cases; RD, rediscussed cases.

The number of discussed cases was 795 in the pre-covid period with a median of nine cases per meeting, 501 cases with a median of 10 cases per meeting in the Covid period, and 710 with a median of six cases per meeting in the post-covid period. The proportion of new discussed cases as compared to the re-discussed ones (Figure 2B) raised from 30.1% (239 cases) in the pre-covid period, to 37.9% (190) in the covid period, and 42.3% (300) in the post-covid period (*p* < 0.0001).

## 4 Discussion

With the onset of the COVID-19 pandemic, telemedicine has rapidly spread throughout the medical field in various contexts, from remote consultations to conferences and online meetings. Teleconsultation between professionals via validated platforms will be the virtual mode of interaction between predefined national reference centres (all of which are already EURACAN centres and so-called “provider centres”) and local centres (so-called “user centres”) within the Italian National Rare Tumors Network (RNTR) (25). In this Network, which is currently being implemented and is in the pilot phase in some Italian regions, clinical cases can be discussed at a multidisciplinary level to ensure the correct diagnostic and therapeutic approach, avoiding unnecessary travel for patients as far as possible.

Virtual DMT for musculoskeletal oncology care has been reported, following the pandemic, as a valuable and effective tool with the aim to simplify a satisfying interaction between specialists (26). Similarly, to other few experiences reported in literature, the results of our survey showed that virtual DMT gives the possibility to break the geographical barriers. It can optimize the work because of the lack of need for a fixed place of meeting increases the flexibility of the work, with no additional travel requirements, which is a factor that may improve attendance (27).

In fact, our results have shown that with the virtualization of the meetings, the attendance was similar or even increased with a median participation that shifted from 58.0% in the pre-covid period to 64.0% in the post-covid one. While in the pre-covid period we observed occasionally the participation of external guests and no one in the covid period, in the post-covid period we registered 11 external guests in total with almost always one guest at every meeting. From our point of view, this data is important, as the participation of external guests, facilitated by the possibility of connecting online, allows for cooperation in the management of patients in the community, and is especially important for a rare disease such as sarcoma, where it is essential that patients are referred to specialist centres. The experience reported shows an increase in the number of meetings from pre-covid period to the post-covid one (from 84 to 51 to 93 meetings in a year). These numbers dropped during the covid period due to the temporary interruption of DMT meetings before the introduction of the virtual modality. A possible reason for the rise in the post-covid period was the gradual recovery of clinical activity and the reduction of sanitary limitations, but it could be linked also to a more organized and simple way of organizing meetings.

From the results of the survey, the introduction of virtual modality has not had any impact on the depth or attention to discussion but rather improved it and there was not a change in treatment plan. The survey results show that 27.3% considered a disadvantage to only be able to participate on site. Twenty-two point 7 % of participants perceived difficulties in interacting and sharing data, and 36.4% believed that image resolution was poorer in the FTF DMT. On the contrary, 90% of participants were highly or moderately satisfied with the technological support provided by the virtual mode. Ninety-one percent of survey participants believed that virtual DMT could be approved in the future, and all participants agreed that the virtual format could encourage the expansion of meetings. Reflecting the survey results, DMT continues to be virtual in our institute, despite the fact that there are no longer any pandemic restrictions. Furthermore, since 2021, our institute has implemented online platforms for recording discussed cases and the attendance of healthcare professionals. As proof that virtual DMT is a valid method for discussing cases, over 800 cases were discussed in 2024.

The survey presented in this article was proposed to assess the degree of appreciation within the multidisciplinary working group dedicated to the treatment of sarcomas at our institute. For this reason, the number of participants is small, that is the principal limitation of this study. Another limitation of the study is that the survey was designed and evaluated by members of the same working group to which it was then proposed, and was not subjected to a validation process.

## 5 Conclusion

Five years after the COVID-19 spread, literature data regarding vDMT meetings remains scarce. Results from this experience showed that the vDMT have been accepted by healthcare professionals with high satisfaction level, due to a better quality of clinical approach/research, technological innovations, and especially logistical setting. In fact, analysing data from institutional DMT we observed an increase in participation rate.

Results from this experience should be extended to all institutional DMT meetings and/or to other reference centres for the treatment of sarcomas. Given the results of our survey, the implementation of virtual DMT or, alternatively, a hybrid form combining in-person and virtual meetings, could become the standard.

## Data Availability

The raw data supporting the conclusions of this article will be made available by the authors, without undue reservation.
